# Applications of Discrete Synthetic Macromolecules in Life and Materials Science: Recent and Future Trends

**DOI:** 10.1002/advs.202004038

**Published:** 2021-01-25

**Authors:** Resat Aksakal, Chiel Mertens, Matthieu Soete, Nezha Badi, Filip Du Prez

**Affiliations:** ^1^ Polymer Chemistry Research Group Centre of Macromolecular Chemistry (CMaC) Department of Organic and Macromolecular Chemistry Ghent University Krijgslaan 281 S4‐bis Ghent B‐9000 Belgium

**Keywords:** data storage, discrete macromolecules, sequence defined polymers, structure–property relationships

## Abstract

In the last decade, the field of sequence‐defined polymers and related ultraprecise, monodisperse synthetic macromolecules has grown exponentially. In the early stage, mainly articles or reviews dedicated to the development of synthetic routes toward their preparation have been published. Nowadays, those synthetic methodologies, combined with the elucidation of the structure–property relationships, allow envisioning many promising applications. Consequently, in the past 3 years, application‐oriented papers based on discrete synthetic macromolecules emerged. Hence, material science applications such as macromolecular data storage and encryption, self‐assembly of discrete structures and foldamers have been the object of many fascinating studies. Moreover, in the area of life sciences, such structures have also been the focus of numerous research studies. Here, it is aimed to highlight these recent applications and to give the reader a critical overview of the future trends in this area of research.

## Graphical abstract

A comprehensive overview of the applications of discrete synthetic macromolecules in biology and materials science is presented. Topics as diverse as macromolecular data storage, self‐assembly, foldamers, catalysis, lectin‐binding, and antimicrobial properties are presented. Additionally, features such as the influence of sequence regulation on the physical properties of such uniform macromolecules are elaborated and future trends in the field are provided.

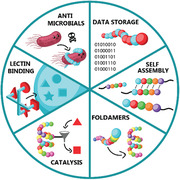

## 1 Introduction

Polymers are nowadays present everywhere in our everyday life.^[^ ^1^ ^]^ Since the implementation of the first fully synthetic plastic by Leo Baekeland in 1907, this area of research has extensively grown. New polymers were created and their performances were continuously improved.^[^ ^2^ ^]^ In parallel, polymerization techniques were developed and enhanced with the aim of gaining control over, e.g., the average molecular weight (*M*_n_), dispersity (*Đ*), architecture or functionality of the formed polymers.^[^ ^3^, ^4^ ^]^ However, the 21st century has come with new challenges and new areas of research have emerged such as the one of sequence‐controlled polymers.^[^ ^5^, ^6^ ^]^ This term has been used to describe polymers in which the monomer sequence is regulated and the dispersity reduced.^[^ ^7^ ^]^ However, the control over the sequence or the polymer length is not necessarily absolute and the properties of the obtained polymer can be affected by the inherent molecular weight distribution.^[^ ^8^, ^9^, ^10^ ^]^

Inspired by advanced technologies found in nature, notably by the functioning of biomacromolecules such as DNA or proteins, polymer chemists are currently looking for more precision chemistry tools in order to prepare synthetic uniform macromolecules with unprecedented properties. As a consequence, the field of “sequence‐defined polymers” has been created. This strong search for ultraprecise, discrete (i.e., strictly monodisperse), synthetic macromolecules led to the development of new synthetic approaches or the reuse/adaptation of already established chemistry tools for their preparation.^[^ ^11^, ^12^, ^13^ ^]^ One of the most widely spread methods is solid phase synthesis.^[^ ^14^ ^]^ Although this technique was initially developed for peptide synthesis, it was since then applied extensively for the formation of discrete synthetic oligomers.^[^ ^15^, ^16^, ^17^, ^18^ ^]^ As a consequence, many chemical reactions, usually performed in solution, have been translated to solid phase synthesis, such as copper‐catalyzed azide‐alkyne cycloadditions, Passerini three component reactions or thiolactone chemistry.^[^ ^19^, ^20^, ^21^, ^22^ ^]^ The interest for solid phase synthesis is driven by the ease of purification, however, it shows some drawbacks too. For example, the chain length can be limited because of incomplete coupling steps or aggregation of the oligomers, thus limiting the accessibility of the chain‐ends, etc.^[^ ^23^ ^]^ Solution phase synthesis approaches are therefore preferred by some research groups.^[^ ^24^, ^25^ ^]^ The reaction can be performed with the help of a soluble polymer support^[^ ^26^, ^27^, ^28^ ^]^ or it can be completed directly in solution.^[^ ^23^, ^29^ ^]^ Additionally, some methods in solution offer the advantage of being up‐scalable.^[^ ^25^, ^30^, ^31^ ^]^

**Figure 1:**
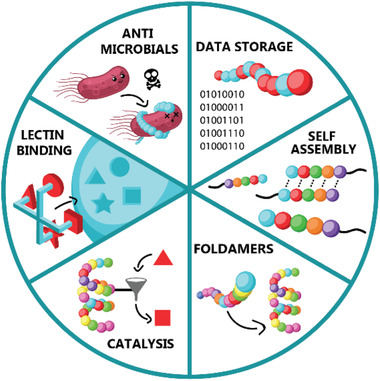
A schematic representation of the applications discussed within this review.

As research articles and reviews dedicated to the development of synthetic routes toward the preparation of uniform macromolecules, have succeeded one another in the last decade, this review will rather focus on their applications in the area of material and life science.^[^ ^8^, ^11^, ^22^, ^32^ ^]^ Hence, in a first part, macromolecular self‐assembly, catalysis and advanced technologies such as macromolecular data storage will be critically examined (**Figure** **1**). In the second part, biologically relevant, precisely engineered macromolecules will be elaborated for their potential lectin binding or antibacterial activity. Other applications such as the formation of duplexes and foldamers will also be discussed.

Although the synthesis of discrete synthetic polymers is not the main focus, a selection of relevant chemistries will be highlighted along the sections of this report. Moreover, peptides and peptidomimetic structures inspired by natural products will not be further discussed in detail here. On the other hand, certain examples will be highlighted where a backbone is used that was initially explored for its peptidomimetic features, but later found its way toward other (non‐biological) areas.^[^ ^33^ ^]^

## 2 Chain Length Limitation and Upscalability Aspects of Discrete Synthetic Macromolecules

This review will highlight some of the areas where discrete synthetic macromolecules have been applied over the last years, yet it should first be stressed that all individual applications have different prerequisites regarding scalability and the chain length of the individual macromolecules. This means that the nature of the desired application should always be considered, when designing a novel synthetic pathway to yield tailor‐made uniform macromolecules. Indeed, certain applications cannot be targeted without a large amount of product and/or long macromolecular chains, while others require only milligram amounts of oligomeric structures. Therefore, this section will feature a concise discussion of some approaches that have either the potential to be scaled‐up or result in long polymer chains, whilst the reader is referred to elsewhere for an elaborate overview of the different synthetic methodologies.^[^ ^8^, ^11^, ^32^ ^]^

One commonly used approach relies on the iterative coupling of building blocks and can be directly performed in solution or by making use of a (soluble) solid support. While the latter offers many advantages for the development of discrete macromolecules (e.g., the purification is only limited to filtration, an excess of reagents can be used to increase the conversion as well as the reaction kinetics, possible automation), the milligram scale reactions in a research lab environment limit the applications to certain specific areas of research. However, although limited to a small number, some research studies demonstrated that higher scales or longer macromolecules can effectively be obtained via support‐based strategies.^[^ ^34^, ^35^, ^36^ ^]^ For example, Fuzeon, a peptide consisting of 36 amino acids, is synthesized annually on a multi‐ton scale by using a combination of solid‐ and solution phase approaches. This macromolecule, although containing a biological backbone, demonstrates that scalability issues could in principle be overcome.^[^ ^37^ ^]^ On the other hand, iterative protocols in solution have less constraints regarding the reaction scale, yet often require the use of labor‐intensive purification steps such as column chromatography.^[^ ^38^ ^]^ Remarkably, Meier and co‐workers reported that, for their specific protocol, the purification of discrete synthetic macromolecules through column chromatography was actually simplified once the chain length increased.^[^ ^25^ ^]^ This rather counterintuitive observation stems from the fact that the excess of low molecular weight reagents could be readily eluted by making use of an apolar solvent, followed by collection of the desired oligomer. In a slightly similar approach, Gao and co‐workers used a polar‐inversed strategy to selectively precipitate their uniform, positively charged macromolecules in the presence of the unreacted neutral monomers, thereby circumventing tedious work‐up steps.^[^ ^29^ ^]^ In another report, Livingston and co‐workers combined a liquid phase approach with selective molecular sieving, which enabled the synthesis of uniform polyethers through simple sieving and extraction steps.^[^ ^24^ ^]^ Very recently, Porel and Alabi demonstrated that a clever choice of building blocks could limit the purification process to a straightforward aqueous extraction.^[^ ^28^ ^]^ This strategy resulted in the multigram synthesis of monodisperse macromolecules, which were eventually used for the synthesis of polymer networks using thiol‐ene chemistry. To our knowledge, the latter manuscript is the first one that introduces sequence‐defined oligomers in a bulk material.

While these studies indicate that the potential of discrete macromolecules should not be hampered by scalability issues, the length of the oligomers remains rather limited.^[^ ^39^ ^]^ This drawback could in principle be overcome by switching to an iterative exponential growth (IEG) strategy, whereby the number of repeating units is doubled in each step.^[^ ^40^, ^41^, ^42^ ^]^ Nevertheless, this method provides a reduced control over the primary structure and is therefore mostly attractive for applications where, for example, alternating blocks are targeted. Alternatively, the use of an automated solid phase approach (vide infra) offers the possibility to investigate the maximal attainable chain length.^[^ ^43^ ^]^ For instance, Lutz and co‐workers synthesized uniform macromolecules with a degree of polymerization (DP) higher than 100 monomers through a modified phosphoramidite approach, demonstrating that molecular weights close to commodity polymers could be achieved.^[^ ^44^, ^45^ ^]^

These examples clearly demonstrate that, since recently, scalability and chain length are in principle not a drawback anymore, although the obtained macromolecules will likely not become cost effective and widely available in the near future. On the other hand, these structures can be used as model compounds in order to investigate a number of applications, including, but not limited to those highlighted in this review.

## 3 Applications of Discrete Synthetic Macromolecules in Material Science Context

### 3.1 Macromolecular Data Storage

It has recently been pointed out that the quantity of microchip‐grade silicon will not be able to follow the increasing demand needed for storing all the data that is being created every day, hence researchers are exploring alternatives to current data storage media.^[^ ^46^, ^47^ ^]^ The fact that DNA is one of the options can hardly be seen as a surprise, as nature has optimized the process of storing an entire genome in these biopolymers over the last billion years, and DNA sequencing has been a topic of interest since decades.^[^ ^48^, ^49^, ^50^, ^51^ ^]^ However, some of the inherent drawbacks associated with DNA (e.g., hydrolysis of phosphodiester bonds, limited Shannon capacity,^[^ ^52^ ^]^ expensive and restricted number of building blocks) can be avoided when using sequence‐defined synthetic macromolecules to encode information at the molecular level as a result of the diverse chemical toolbox available.^[^ ^53^ ^]^

**Figure 2:**
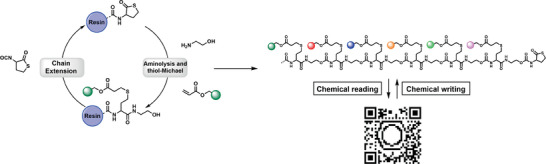
Encoding the information of a QR code into a set of 71 different uniform macromolecules by using an automated amine‐thiolactone‐ene protocol. Adapted under the terms of the CC BY 4.0 license^.[^ ^58^ ^]^ Copyright 2018, The Authors, published by Springer Nature.

Indeed, while DNA sequences consist of four different nucleobases, the number of different repeating units that can be incorporated into sequence‐defined macromolecules is substantially higher, potentially enabling the creation of a far denser data storage media.^[^ ^54^, ^55^ ^]^ This can be exemplified as follows: using a binary alphabet enables the synthesis of 64 different hexamers, while the use of ten different building blocks could already result in 10^6^ different hexamers. This strategy was adopted by Boukis and Meier, who were able to create a “molecular alphabet” with 116 different building blocks by combining two multicomponent reactions. This achievement eventually allowed them to store up to 24 bits of data per repeating unit.^[^ ^56^ ^]^ In a recent report, the same group developed a synthetic approach based on the Passerini three component reactions, where the backbone and the side chain of each individual repeating unit could be varied.^[^ ^57^ ^]^ This dual sequence definition resulted in higher storage capacities compared to more conventional methods due to the increased number of possible permutations. In parallel, Du Prez and co‐workers made use of a similar strategy for the encoding of a QR code onto a set of short sequence‐defined oligomers.^[^ ^58^ ^]^ In this case, the introduction of fifteen different acrylates in their automated amine‐thiolactone‐ene protocol avoided the need for longer macromolecular chains, an often cumbersome challenge using a solid phase approach, to store a vast amount of information (**Figure** **2**).^[^ ^20^ ^]^

Alternatively, an increase in data storage capacity can also be accomplished via the synthesis of longer uniform macromolecules.^[^ ^44^, ^59^, ^60^ ^]^ Moreover, different examples exist that ease the storage and the read‐out of the encoded information of those long macromolecules based on a smaller molecular alphabet (i.e., binary code).^[^ ^61^, ^62^ ^]^ While these approaches require a high synthetic effort, it has already been shown that uniform macromolecules with a DP of 100 could be attainable when using a solid support.^[^ ^44^ ^]^ Despite the fact that longer sequences were so far only reported with support‐assisted strategies, the study of Lee et al. clearly exemplified that other synthetic approaches are also capable of yielding longer, uniform macromolecules.^[^ ^63^ ^]^ They demonstrated the scalable synthesis of a binary encoded, high molecular weight copolyester (up to 38 kDa) by making use of an elegant cross‐convergent pathway, combined with preparative size exclusion chromatography as an (automated) purification method.

While storing data onto the molecular level is already an accomplishment, an accurate and error‐free read‐out of the information (referred to as sequencing) is of utmost importance. Most research groups rely on tandem mass spectrometry techniques to determine the order of the different monomeric blocks. These techniques are often accompanied by in‐house developed algorithms, as it also is commonly used in the field of proteomics.^[^ ^64^, ^65^ ^]^ In addition, the structures of the macromolecules can be designed in a specific manner to facilitate the straightforward retrieval of the encoded data. In this context, Lutz and co‐workers showed that the MS/MS read‐out of acid‐terminated encoded polyurethanes was significantly simplified when a H/Na exchange occurred at the acid‐end, especially for structures with a high DP.^[^ ^66^ ^]^ On the other hand, the same group tuned the synthesis of a uniform poly(alkoxyamine phosphodiester) in such a way that the encoded data could not be unambiguously retrieved anymore when making use of MS/MS techniques.^[^ ^67^ ^]^ Instead, the data could only be revealed when making use of ion mobility spectrometry measurements, which revealed a key that was necessary to allow actual decryption via MS/MS, opening possibilities in the area of anti‐counterfeiting applications (vide infra). Furthermore, Zhang and co‐workers demonstrated that the MS/MS analysis of uniform macromolecules based on a thiol‐maleimide coupling could tremendously be enhanced by oxidizing the thioether to a sulfoxide.^[^ ^68^, ^69^ ^]^ This resulted in a decreased bond dissociation energy of the carbon—sulfur bond, which allowed a selective chain cleavage during the sequencing process.

**Figure 3:**
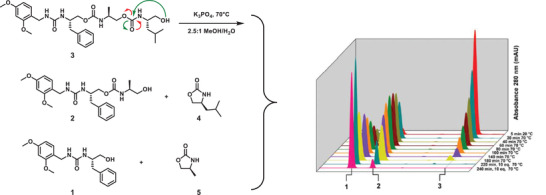
Sequencing of sequence‐defined oligourethanes (3) through a self‐immolative process (depicted with green arrows) combined with LC–MS traces at given intervals. Adapted with permission.^[^ ^73^ ^]^ Copyright 2020, American Chemical Society.

On the other hand, verification of the monomer order can, in principle, be achieved by other approaches such as NMR (although the analysis of long sequences might be challenging) or nanopore sequencing.^[^ ^70^, ^71^ ^]^ While the latter demonstrated a great potential for the structure elucidation of DNA, to date, no report describing the successful sequence analysis of synthetic uniform polymers using this technique could be found in literature.^[^ ^72^ ^]^ On the other hand, Anslyn and co‐workers recently reported the structure determination of oligourethanes via a controlled depolymerization process, which exhibited some resemblance to Edman degradation, a well‐known technique used during the sequencing of peptides.^[^ ^73^ ^]^ These self‐immolative macromolecules possess a terminal alcohol moiety that is used as a functional handle during an intramolecular cyclisation process, given the oligomers are placed under appropriate conditions (i.e., basic medium and microwave irradiation), thereby yielding a cyclic monomer together with the depolymerized chain (**Figure** **3**). Interestingly, performing one single liquid chromatography mass spectrometry (LC–MS) analysis after a given reaction time allows the detection of all possible intermediates (i.e., monomer, dimer, trimer, etc.), hence enabling the read‐out of the encoded information. In a similar case, Lutz and co‐workers reported the degradation of encoded poly(N‐substituted urethane)s in a basic medium through the same unzipping mechanism, yet degradation could already be achieved at milder conditions.^[^ ^74^ ^]^

Currently, the speed of writing and reading protocols applicable to biopolymers and uniform synthetic macromolecules is still far from the one of silicon‐based storage media. Hence, viable applications are mainly those focusing on archival data storage purposes (i.e., stored for an extended period of time and a delayed read‐out). Nevertheless, it has already been demonstrated that synthetic macromolecules could also function as molecular barcodes in anti‐counterfeiting applications,^[^ ^75^, ^76^, ^77^ ^]^ an area of research in which the need for additional protective measures is of high interest.^[^ ^78^ ^]^ In this context, Du Prez and co‐workers recently reported the preparation of “macromolecular pin codes.” The introduction of dynamic covalent bonds into their oligomers offered the possibility to scramble and encrypt encoded data, hence adding another layer of security to anti‐counterfeiting tags. They further demonstrated the real‐life applicability of their concept by depositing the encrypted oligomers on plastic banknotes, followed by an extraction and the subsequent read‐out by means of electrospray ionization mass spectrometry (ESI–MS).^[^ ^79^ ^]^ Deciphering the encoded data could only be successfully achieved when the reader was in possession of a “key”, which was necessary to assign the different isotopic patterns of the monomeric units to their actual position in the sequence. In a similar context, Lutz and co‐workers described the development of different sequence‐encoded poly(phosphodiesters).^[^ ^80^ ^]^ In their study, the encoded information could be altered by subjecting the macromolecules to photo‐irradiation. In one example, the macromolecules were composed of light‐sensitive and light‐inert moieties that had identical masses (*o‐* and *p‐*nitrobenzyl), thus MS/MS analysis was not able to differentiate between both monomers and the encoded data could only be revealed once the sequence had been exposed to light. Another approach has been proposed by Zhang and co‐workers who showed that data could be stored in more complex molecular architectures such as dendrimers.^[^ ^81^ ^]^ In this case, the complicated MS/MS spectra cannot directly deliver the encoded information since a rule of encryption, that can be designed and changed on demand, is necessary to obtain the unique data matrix for a certain dendrimer.

The examples described in this section clearly indicate that uniform synthetic macromolecules could play a crucial role in many aspects of future data management. Even though the number of studies focusing on the use of information encoded molecules is increasing rapidly,^[^ ^82^ ^]^ their enormous potential has not yet been fully harnessed. Indeed, we are confident that this report and others will spark the interest in the development of novel approaches that will further revolutionize the contemporary perception of data storage and encryption.^[^ ^83^ ^]^

Besides data storage applications, discrete synthetic macromolecules can be used to induce selective self‐assembly into complex structures. The next section will focus on the highlights in this area of research.

### 3.2 Self‐Assembly of Discrete Synthetic Macromolecules

#### 3.2.1 Self‐Assembly of Discrete Block Copolymers

Interactions between different molecules to form higher‐ordered structures are omnipresent in nature. Typical examples are the congregation of phospholipids to form the cellular membrane or the assembly of numerous copies of coat proteins in the outer shell of viruses, which give them their distinct shapes.^[^ ^84^, ^85^ ^]^ The molecular assembly of small amphiphilic molecules has been studied extensively and theoretical models have been developed to rationalize and predict their behavior based on the molecular geometry and supramolecular interactions.^[^ ^86^ ^]^ Also block copolymers can show the tendency to assemble into well‐defined morphologies. This phenomenon is a result of the incompatibility between the different blocks, which segregate to minimalize the interfacial energy. Similarly, theoretical and computational models have been developed to describe this.^[^ ^87^ ^]^ However, these models often assume discrete block copolymers, in contrast to the disperse nature of the actual polymers used in experimental studies, which are obtained via different living and controlled polymerization strategies.^[^ ^87^, ^88^ ^]^ Therefore, these models do not grasp the complete picture and do not account for variation in the chain length. Nevertheless, the continuous evolution of the field of sequence‐controlled polymers has nowadays enabled the synthesis of discrete counterparts of typical disperse commodity polymers.^[^ ^89^, ^90^, ^91^, ^92^, ^93^, ^94^, ^95^, ^96^, ^97^, ^98^ ^]^ Recent reports by the groups of Meijer and Hawker have already indicated that even a very low dispersity can have a dramatic influence on the self‐assembly properties of block copolymers in the low molecular weight regime.^[^ ^99^, ^100^ ^]^

**Figure 4:**
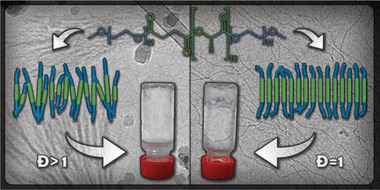
Discrete ABA‐type block copolymer with ethylene glycol and (l)‐lactic acid blocks studied by Meijer and co‐workers formed a transparent gel in water. Introduction of chain length variation (*Đ* = 1.2) in the (l)‐lactic acid block inhibited the self‐assembly and led to solubility under the same conditions. Adapted with permission.^[^ ^9^ ^]^ Copyright 2018, American Chemical Society.

For example, uniform dimethylsiloxane‐lactic acid copolymers were reported by Meijer and co‐workers.^[^ ^101^ ^]^ In their work, a synthetic strategy was developed for the synthesis of discrete oligodimethylsiloxane, while the synthesis of discrete atactic poly(lactic acid) blocks was adopted from an earlier report.^[^ ^94^ ^]^ Both blocks were first synthesized separately and conjugated in the final step. The combination of these constituting blocks was previously already investigated in disperse block copolymers, which revealed a high *χ*‐parameter (i.e., a high incompatibility between the blocks).^[^ ^102^, ^103^, ^104^, ^105^ ^]^ Different discrete block copolymers were investigated with a molecular weight below 7 kDa, while the volume fraction of lactic acid was varied between 0.25 and 0.50. Only the samples with a low fraction of lactic acid were disordered, while the others showed distinct bulk morphologies. Moreover, these discrete copolymers were compared to a disperse reference sample with a similar average molecular weight and composition. Surprisingly, the disperse reference did not show any evidence for an ordered structure. The influence of low levels of dispersity was further investigated for this system, which revealed a few trends. Introducing a low dispersity (*Đ* < 1.10), lead to a widening in domain spacing and a lower overall degree of ordering compared to the discrete block copolymer.^[^ ^99^, ^100^ ^]^ In contrast, the order–disorder transition shifted toward higher temperatures for the disperse samples. Therefore, uniformity seems to be a promising tool to design materials with small feature sizes.^[^ ^105^ ^]^ The same observations were made for poly(dimethylsiloxane‐*b*‐methyl methacrylate) block copolymers by the group of Hawker.^[^ ^100^ ^]^ The importance of uniformity was further highlighted, when the amorphous atactic lactic acid block was changed to (l)‐lactic acid, which can form crystalline domains. This ability to form crystalline domains leads to a high uniformity in the domain spacing of the discrete block copolymers. However, the degree of ordering was dramatically decreased when dispersity was introduced in the crystalline block.^[^ ^99^, ^106^ ^]^ Also in other studies, the influence of the backbones’ tacticity was found to have a significant effect on the non‐covalent interactions between oligomers.^[^ ^43^ ^]^ While the previous examples illustrate the influence of dispersity in bulk, a remarkable difference in solution was reported for ABA‐type block copolymers with ethylene glycol and (l)‐lactic acid blocks by Meijer and co‐workers.^[^ ^9^ ^]^ Indeed, the discrete block copolymer formed a transparent gel in water, while the disperse counterpart, with similar composition and average molecular weight, was soluble in the same conditions (**Figure** **4**).

Johnson and co‐workers developed an iterative exponential growth strategy for the synthesis of stereo‐controlled sequence‐defined macromolecules.^[^ ^40^, ^41^ ^]^ Their approach is based on the Cu(I)‐catalyzed azide‐alkyne cycloaddition to couple different fragments, combined with the epoxide ring‐opening using an azide anion to introduce this moiety. In addition, they demonstrated the ability to introduce moieties via radical thiol‐ene chemistry. Using this approach, uniform block copolymers were obtained.^[^ ^40^ ^]^ While these consisted of the same backbone, the difference in side chain moieties between the blocks enabled microphase separation. The first block was functionalized with decane side chains, while the other block was made hydrophilic by the introduction of either a glycerol‐ or triethylene glycol monomethyl ether group. Small‐angle X‐ray scattering measurements showed a hexagonal cylinder morphology, which was further supported by transmission electron microscopy images. Differential scanning calorimetry indicated an amorphous nature of the self‐assembled material. In contrast, the self‐assembly of a similar peptoid‐based system was driven by crystallization.^[^ ^107^ ^]^

In summary, while examples of discrete block copolymers are still scarce, the highlighted ones already show that uniformity plays an unprecedented role in low molecular weight macromolecular structures. Introduction of even modest chain length resulted in remarkable differences.

#### 3.2.2 Sequence‐Selective Assembly and Duplex Formation of Precision Oligomers

**Figure 5:**
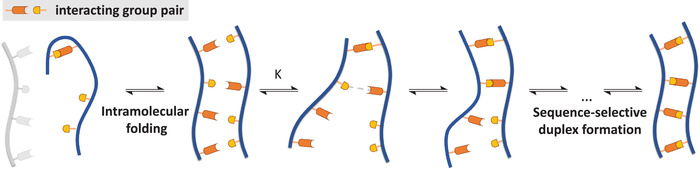
Duplex assembly process between two hetero‐oligomers bearing a complementary sequence of interacting groups. Adapted with permission.^[^ ^129^ ^]^ Copyright 2017, American Chemical Society.

Biomacromolecules with a defined monomer sequence form the basis of life as we know it.^[^ ^108^ ^]^ Prediction of the folded protein structure from the amino acid sequence remains challenging, despite the significant progress that has been made in the last decades.^[^ ^109^ ^]^ In contrast, nucleic acids possess one of the most predictable and programmable interactions due to the Watson–Crick base pairing.^[^ ^110^ ^]^ The predictability of this base pairing gave rise to DNA nanotechnology and was used to form complex nanoscale structures by assembly of nucleic acid chains, through careful and rational design of the sequence.^[^ ^111^, ^112^, ^113^ ^]^ Synthetic nucleic acid analogues have been developed, demonstrating that these characteristics are not limited to the sugar–phosphate backbone of DNA.^[^ ^114^, ^115^, ^116^, ^117^, ^118^ ^]^ In theory, every macromolecule equipped with complementary recognition units, has the potential to form sequence‐selective duplexes. Duplex formation has been achieved using hydrogen bonding^[^ ^119^, ^120^, ^121^, ^122^, ^123^, ^124^ ^]^ or metal–ligand^[^ ^125^, ^126^, ^127^, ^128^ ^]^ interactions by different research groups. However, in these examples the interacting groups are often embedded in the backbone and the influence of the sequence was not always studied. In this paragraph, recent examples that apply a more modular approach, where the interacting groups are introduced as side chains similar to nucleic acids, will be discussed (**Figure** **5**).

Two oligomers containing complementary interacting groups can assemble into a bimolecular duplex. The different equilibria involved in this process are represented in Figure 5.^[^ ^129^ ^]^ When two non‐bonded oligomers are in close proximity, a first interaction can be formed between the complementary groups. The intrinsic strength of this interaction is dependent on the choice of the groups and is associated with the intramolecular association constant (*K*). After this first interaction, the system can either disassemble or further “zip‐up” to form the duplex. The assembly often occurs in a cooperative fashion. In the case of hetero‐oligomers, i.e., an oligomer that contains both groups of the interacting pair, intramolecular folding can compete with the duplex formation. By imposing conformational strains on the backbone, this competing pathway can be reduced or eliminated.

**Figure 6:**
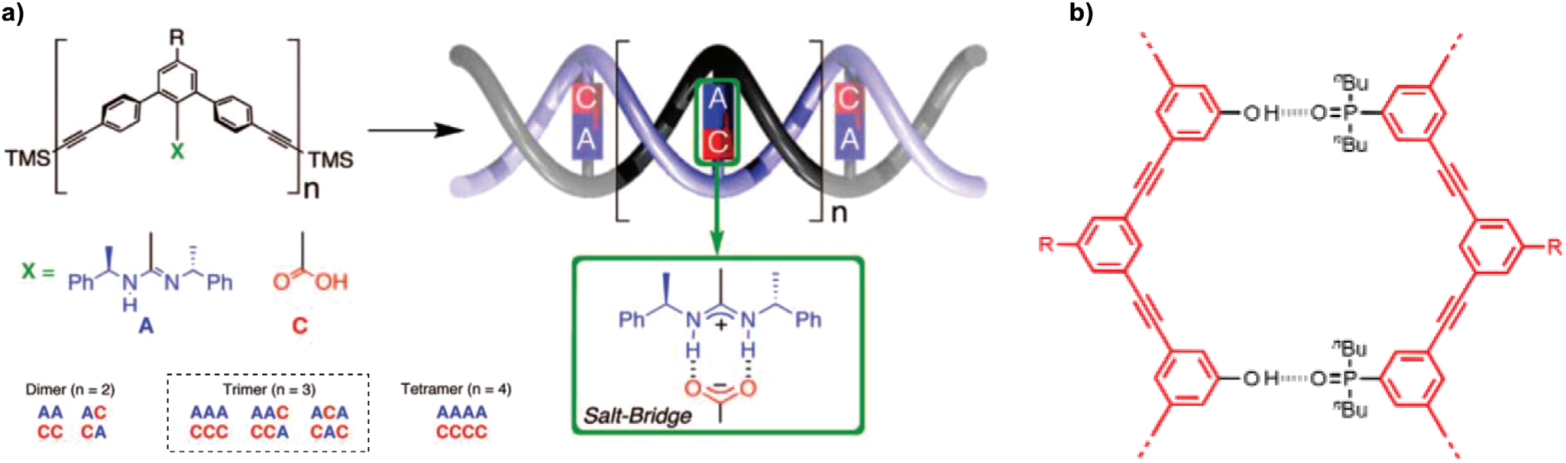
a) Structure of *m*‐terphenyl–diacetylene oligomers bearing amidine (A) and carboxylic acids (C) moieties that form saltbridges. Adapted with permission.^[^ ^130^ ^]^ Copyright 2008, American Chemical Society. b) Structure of duplex‐forming phenylacetylene oligomers with phenol as hydrogen bond donor and phosphine oxide as hydrogen bond acceptor. Adapted with permission.^[^ ^131^ ^]^ Copyright 2018, American Chemical Society.

Rigid oligomers with a *m*‐terphenyl–diacetylene backbone that form double helices have been described by Yashima and co‐workers.^[^ ^130^ ^]^ The duplex formation occurs through salt bridges between amidine and carboxylic acid moieties (**Figure** **6**a, A and C, respectively), which are attached to the terphenyl group of the backbone. Due to the rigidity of the latter, hetero‐oligomers can be prepared without the risk for intramolecular folding. The sequence‐selectivity of the duplex formation was assessed by mixing two homo‐oligomers with either two amidine moieties (AA) or two carboxylic acids (CC) and a self‐complementary oligomer (AC). This led to the formation of two duplexes, namely the heteroduplex [AA/CC] and the homoduplex [AC/AC]. A similar type of duplex‐forming oligomers with a rigid phenylacetylene backbone was reported by Hunter and co‐workers (Figure 6b).^[^ ^129^, ^131^ ^]^ In this case, formation of H‐bonded duplexes was possible by the interaction of a phenol and a phosphine oxide moiety as hydrogen bond donor and acceptor, respectively. This resulted in very stable duplexes, with an association constant of 10^8^ m ^−1^ in toluene for a pair of tetramers.

**Figure 7:**
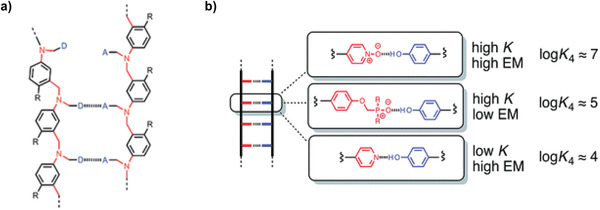
a) Backbone structure of duplex‐forming tetramers containing hydrogen bond donors (D) and acceptors (A). b) Different acceptor and donor pairs with the association constant for the tetrameric duplexes. Adapted with permission.^[^ ^132^, ^134^ ^]^ Copyright 2016, The Royal Society of Chemistry.

Hunter and co‐workers have also investigated oligomers with a more flexible backbone.^[^ ^132^, ^133^ ^]^ Strong hydrogen bond acceptors (e.g., phosphine oxide, pyridine oxide and pyridine) were used in combination with phenol as hydrogen bond donor (**Figure** **7**). To study the influence of the donor and acceptor pairs on the duplex stability, homo‐oligomers (i.e., bearing only hydrogen bond donors or acceptors) were prepared. Although the intrinsic strength of the H‐bonds formed between phenol and corresponding acceptors (phosphine oxide or pyridine oxide) are in the same order in toluene (*K* ≈ 315 m ^−1^), the association constant was found to be lower when combining phenol and pyridine (*K* ≈ 31 m ^−1^). Remarkably, the tetrameric duplex formed with pyridine oxide was found to be more stable than the one obtained with phosphine oxide (Figure 7b). This effect was assigned to the higher conformational freedom of the latter, illustrating that the stability of the duplex is not solely dependent on the intrinsic strength of the interacting groups and the nature of the backbone.

However, contrary to what is observed for homo‐oligomers, it was demonstrated that a flexible backbone could be detrimental for oligomers bearing both hydrogen bond donors and acceptors, as intramolecular folding can compete with the formation of the duplex.^[^ ^135^, ^136^, ^137^ ^]^

**Figure 8:**
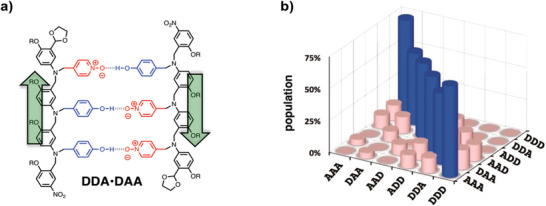
a) Structure of a hydrogen bonded duplex formed with two sequence‐complementary oligomers in an antiparallel orientation. b) Calculated populations of the duplexes formed in toluene when all oligomers are mixed in equimolar ratios. Adapted with permission.^[^ ^138^ ^]^ Copyright 2017, The American Chemical Society.

In another study, the folding of short dimers with different backbones and the aforementioned hydrogen bond acceptors was also investigated.^[^ ^129^ ^]^ An AD‐type oligomer containing phosphine oxide as acceptor showed a significant fraction in the folded state (≈69%). Remarkably, when the acceptor was changed to pyridine oxide, the folding was not observed anymore. Compared to phosphine oxide, these moieties are closer to the backbone, reducing the overall flexibility. As a 1,2‐folding did not occur in these type of oligomers, all possible trimer combinations were prepared to explore the possibility of sequence‐selective duplex formation (**Figure** **8**).^[^ ^138^ ^]^ The interaction between all pairs of oligomers were investigated, including mismatched sequences. When an acceptor was changed with a donor (e.g., A → D mutation), more stable complexes were obtained compared to the opposite D → A mutation. This effect was attributed to the ability of the acceptor to be involved in multiple hydrogen bonds, increasing the stability. It was also found that a 1,3‐folding lead to competition with the duplex formation.

A final factor that contributes to the duplex stability is the orientation of the backbone, whereby an antiparallel orientation was found to be around four times more stable. The association constants were used to calculate the population of each possible duplex when all the different oligomers are mixed (Figure 8b), which demonstrates that the duplexes formed with sequence‐complementary oligomers are the most abundant.

In the previous examples, non‐covalent interactions between different oligomers resulted in the formation of molecular duplexes. The dynamic nature of these bonds provided pathways for error correction of mismatched structures, allowing the oligomers to rearrange until the thermodynamic equilibrium is reached. Alternatively, dynamic covalent chemistry can also provide similar pathways for correction, which has already been used for the assembly of larger structures.^[^ ^139^ ^]^

**Figure 9:**
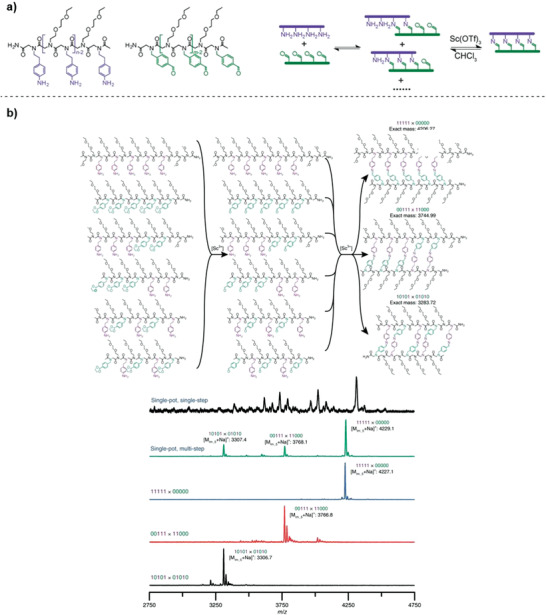
a) Structure of amine‐ and aldehyde‐bearing homo‐oligomers and scheme of the duplex formation. Reproduced with permission.^[^ ^140^ ^]^ Copyright 2015, The American Chemical Society. b) (Top) Sequence‐selective duplex formation of several pairs of complementary oligomers using the dissociation/extraction/annealing process. (Bottom) matrix assisted laser desorption ionization ‐ time of flight (MALDI) mass spectra of individual encoded molecular ladders assembled via the dissociation/extraction/annealing process, including 10 101 × 0 1010 (bottom, black), 00 111 × 11 000 (second from bottom, red), 11 111 × 00 000 (middle, blue), and a single‐pot solution of all six oligomers to yield three in‐registry molecular ladders (second from top, green). A single‐pot solution of the six oligomers after the single‐step, deprotection and direct assembly process (top, black) is shown for comparison. Reproduced with permission.^[^ ^142^ ^]^ Copyright 2020, Springer Nature.

Scandium(III)‐catalyzed imine metathesis was employed by Scott and co‐workers to assemble duplexes in a sequence‐selective manner.^[^ ^140^, ^141^, ^142^ ^]^ A peptoid backbone was chosen, because of the efficient synthesis using the submonomer strategy and the ability to introduce side chain moieties in a straightforward fashion. As peptoids can adopt a Σ‐strand conformation whereby consecutive groups are presented on opposite sites, the aldehyde and amine moieties that form the imine bond were alternated with an inert group (**Figure** **9**a).^[^ ^140^, ^143^ ^]^ Amine‐ and aldehyde‐bearing homo‐oligomers of the same length were mixed with a catalytic amount of scandium(III) triflate, initially forming imine bonds that result in misaligned duplexes. Due to the dynamic nature, these bonds can reshuffle until a duplex is formed. Duplexes with up to six imine bonds were successfully formed, while in a similar study with *m*‐phenylene–ethynylene based oligomers, kinetically trapped duplexes were observed beyond four imine bonds.^[^ ^144^ ^]^

Mixed sequence oligomers, containing both amine and aldehyde groups were investigated in a later study.^[^ ^142^ ^]^ Whilst the imine metathesis was efficient in reshuffling misaligned duplexes, sequence‐selective hybridization required the exchange of mismatched oligomers (e.g., noncomplementary sequence). When three pairs of sequence‐complementary sequences were in situ deprotected followed by direct assembling, a mixture of wrongly assembled structures was obtained.^[^ ^142^ ^]^ Nevertheless, the authors circumvented this by developing a strategy inspired by DNA nanotechnology, where an increase in temperature can dissociate DNA strands to selectively hybridize complementary strands by gradually cooling down. Similarly, by increasing the scandium triflate concentration, the equilibrium could be shifted toward the free amine/aldehyde groups, whereas removal of the catalyst by aqueous extraction resulted in annealed oligomers (Figure 9b).

Moreover the reversible nature of the condensation reaction between 1,2‐diols and boronic acids gained a lot of interest in recent years and was used in applications ranging from biosensors^[^ ^145^ ^]^ to self‐healing materials.^[^ ^146^, ^147^ ^]^ Scott and co‐workers reported the synthesis of phenyl boronic acid and catechol bearing peptoid homo‐oligomers, which formed duplexes with up to six boronic ester bonds.^[^ ^148^ ^]^ Around the same time, this chemistry was also applied by the group of Weil with a peptide backbone.^[^ ^149^ ^]^ In their work, the effect of a mismatched sequence and the introduction of a non‐binding unit on the overall association constant was determined, which suggests possibilities for sequence‐encoded recognition. A poly(ethylene glycol) (PEG_5000_) was attached to a hexamer containing three boronic acid groups, while the complementary diol‐bearing peptide was conjugated to a protein (cytochrome *c*). The conjugation of both entities through duplex formation was confirmed via MALDI–ToF MS, demonstrating possibilities for sequence programming of macromolecules using short oligomeric recognition units.

As already mentioned in this section, besides duplex formation, also folding of the macromolecules can occur. The next section will thus be dedicated to the particular example of foldamers.

### 3.3 Foldamers Based on Uniform Macromolecules

**Figure 10:**
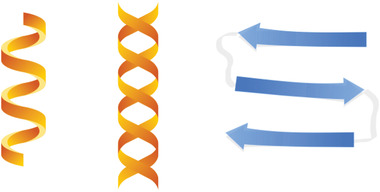
An overview of different architectural motives that are often observed for foldamers; a single helix (left), double helix (middle), and *β*‐sheets (right).

The folding of biomacromolecules into secondary, tertiary, or even quaternary structures enables them to perform a whole range of complex but remarkable functions such as molecular recognition, transportation, information storage, and catalysis, by using only a limited repertoire of building blocks (i.e., 20 amino acids and 4 nucleobases). Moreover, the folding of macromolecules into native states is a fairly delicate process, whereby numerous effects are involved.^[^ ^150^, ^151^, ^152^ ^]^ On the one hand, folding is promoted by favorable enthalpic interactions such as intramolecular hydrogen bonding, electrostatic‐, hydrophobic‐ and van der Waals interactions. On the other hand, creating a secondary structure is associated with locking the degree of freedom and is therefore disfavored in terms of entropy. Thus, folding can be regarded as a balance between different interactions opposing each other, while being a highly cooperative process in most cases. Over the years, many studies have demonstrated that the fundamental principles governing the folding of biomolecules can be extrapolated toward their synthetic counterparts.^[^ ^150^ ^]^ Indeed, different research groups started investigating whether uniform macromolecules based on abiotic building blocks were also capable of adopting specific 3D architectures that could further advance the unique properties exhibited by biomacromolecules. These studies led to the development of synthetic foldamers, a class of molecules that encompasses all structures with an inherent propensity to fold, while incorporating either partially or exclusively non‐natural building blocks.^[^ ^151^, ^153^ ^]^ The latter offers almost unlimited possibilities compared to the 20 amino acids or 4 nucleobases, while the 3D motives of most foldamers show a remarkable resemblance to the ones found in nature (**Figure** **10**).

The first reports on synthetic foldamers were merely focused on making adjustments to the tools that nature offered. Among others, these resulted in the development of peptide nucleic acids (PNAs), a class of achiral peptides that bear nucleobases in the side chain.^[^ ^154^, ^155^ ^]^ In addition, Gellman and co‐workers reported the folding of *β*‐peptides into helical conformations,^[^ ^156^ ^]^ while Zuckermann demonstrated that peptoids, *N*‐substituted glycine oligomers, were able to adopt a polyproline type 1 helix.^[^ ^157^, ^158^, ^159^, ^160^ ^]^ On the other hand, it was also shown that uniform synthetic macromolecules that exhibit little to no resemblance to biopolymers are also able to adopt a secondary structure. Many of these foldamers incorporate aromatic groups (**Table** **1**), a consequence of the fact that these moieties reduce the number of accessible conformations (hence decreasing the entropy loss) and could give rise to *π*–*π* stacking, thereby aiding the oligomers to adopt a stable 3D architecture.^[^ ^161^, ^162^, ^163^, ^164^, ^165^ ^]^ In this context, aromatic oligoamides have emerged as a prominent family of foldamers as a result of their extraordinary properties.^[^ ^151^, ^162^ ^]^ These oligomers have predictable folding patterns in a range of different solvents and retain the helical structure up to temperatures reaching 120 °C in dimethyl sulfoxide. They also provide valuable structural information at the atomic level with their tendency to crystallize. Furthermore, Guichard and co‐workers demonstrated that *N*‐*N*′‐linked oligoureas fold into a stable helix, even in a protic solvent like methanol, as a result of intramolecular hydrogen bonds combined with steric hindrance between the chiral side chain and the N–H present in the main chain.^[^ ^166^, ^167^, ^168^ ^]^ Moreover, many more foldamer systems have already been described, a limited selection of which can be found in Table 1.

**Table 1:** An overview of some relevant foldamers, including their native states combined with the main driving force for adopting the ordered structure

Foldamer	Structure	Native state	Main driving force	Reference
PNA	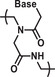	Double helix with complementary DNA or PNA strand	Hydrogen bonding between matching nucleobases	^[^ ^154^, ^155^ ^]^
*β*‐peptides	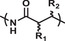	Multiple helical structures	Intramolecular hydrogen bonding	^[^ ^156^, ^170^ ^]^
Peptoids	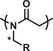	Helices Turns Loops	Restricted number of accessible conformations, *n* → π* interactions	^[^ ^157^, ^171^, ^172^, ^173^, ^174^ ^]^
Benzoyl urea	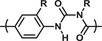	Helical structures	Intramolecular hydrogen bonding	^[^ ^175^ ^]^
Aza‐aromatic oligomers	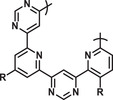	Helical structures	Intramolecular repulsive electrostatic interactions	^[^ ^176^ ^]^
Aromatic oligoamide	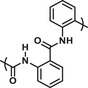	Helical structures *β*‐sheet	Intramolecular hydrogen bonding, restricted number of accessible conformations	^[^ ^162^, ^163^, ^177^ ^]^
*N,N*′‐Oligourea	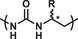	Helical structures	Intramolecular hydrogen bonding	^[^ ^166^ ^]^
Oligo (*ortho*‐arylene)	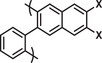	Helical structures	Intramolecular *π*–*π* stacking	^[^ ^161^ ^]^

These examples demonstrate that the first (crucial) hurdle toward the realization of artificial proteins has already been overcome. In addition, backbone heterogeneity (i.e., the polymer sequence) is another important feature that permits to fully harvest the potential of synthetic foldamers, since this could allow them to carry out functions such as molecular recognition and catalysis (Section 3.4). In this context, Huc and co‐workers reported on the selective encapsulation of fructose by using an aromatic oligoamide foldamer.^[^ ^169^ ^]^

Initially, a certain sequence was synthesized that gave rise to a cavity inside the folded oligomer, displaying only little selectivity in binding to a series of monosaccharides. Next, different mutations were introduced in the primary structure that altered the inner cavity in such a way that undesired monosaccharides were excluded due to the increased sterical hindrance, while preserving crucial interactions with the substrate of choice. Remarkably, all induced mutations were introduced by rational design rather than by a screening approach. This was possible since the host–guest complexes afforded crystal structures suitable for X‐ray crystallography, allowing for crucial structural information at the atomic level. Following a series of optimizations in design, the final abiotic foldamer was able to selectively bind fructose in the presence of competing monosaccharides, despite the structural similarities between the different potential guests. In another report, the same group used a similar iterative evolution approach for the preparation of an abiotic foldamer that was capable of selectively binding either tartaric acid or maleic acid, depending on the primary structure.^[^ ^178^ ^]^ These studies beautifully demonstrate that the evolutionary processes can be mimicked by an organic chemist in an extremely short time span, compared to the design of natural proteins over millions of years. Finally, Guichard and co‐workers used a self‐assembled foldamer helix bundle that was composed of six oligourea helices, hence forming a tertiary structure rather than a secondary one, for the encapsulation of different primary alcohols.^[^ ^179^ ^]^ Remarkably, the internal cavity was characterized by a certain degree of flexibility, which means that the volume of the cavity could be adjusted according to the guest present.

Next to molecular recognition and catalysis, the ability to easily introduce a high degree of chemical diversity while maintaining a stable native state is one of the reasons why foldamers have also been investigated as potential therapeutics.^[^ ^180^ ^]^ In addition, the inherent stability of abiotic macromolecules toward proteases is a very attractive feature of synthetic foldamers in the field of drug development.^[^ ^181^ ^]^ Miranker and co‐workers demonstrated the ability of a folded oligoquinoline amide to interact with islet amyloid polypeptide (IAPP).^[^ ^182^ ^]^ Aggregation of the latter results in the formation of amyloid fibers, which have been shown to induce *β*‐cell toxicity.^[^ ^183^ ^]^ The presented foldamer effectively stabilized non‐toxic conformers of IAPP, thereby prohibiting *β*‐cell death. Remarkably, the dynamic character of the foldamers enabled them to cross the cell membrane without any cellular assistance. In another study, Huc and co‐workers developed a series of single helical aromatic oligoamides, one of which perfectly mimicked the charge surface of double‐stranded DNA.^[^ ^184^ ^]^ Furthermore, it was demonstrated that these synthetic uniform macromolecules were able to inhibit different DNA‐binding enzymes. Intriguingly, while the phosphate units play a pivotal role during this process, it is actually the chemical differences, instead of the similarities with DNA that resulted in a higher binding affinity compared to DNA itself. Lastly, the large surface area of foldamers combined with tunable dynamics was shown to potentially inhibit protein–protein interactions, and the use of foldamers as anticancer therapeutics was demonstrated.^[^ ^52^, ^185^, ^186^, ^187^, ^188^, ^189^ ^]^

In summary, foldamer sequences can be tuned through the incorporation of various monomers at specific positions, which enabled the exploration of foldamers as synthetic receptors, novel catalytic systems or potential drug scaffolds.^[^ ^180^, ^190^, ^191^, ^192^ ^]^ Additionally, some groups have recently been focusing on developing higher‐ordered structures (e.g., tertiary and quaternary structures), an endeavor that could further broaden the range of potential applications.^[^ ^193^, ^194^ ^]^ Such reports indicate that foldamers will remain a highly investigated class of molecules in the near future, suggesting that many seemingly impossible challenges could be tackled. We believe that similar studies will eventually pave the way to the development of large discrete synthetic macromolecules that fold into tunable, stable, and predictable native states, and which are able to mimic or even outperform the most sophisticated proteins.

Another application of those discrete structures can be found in the field of catalysis, as highlighted in the examples presented in the next section. With the introductory paragraph dedicated to enzymes, one can see that folding of macromolecules can be linked to their catalytic properties.

### 3.4 Catalysis

One of the most fascinating features of enzymes is their remarkable ability to catalyze all chemical transformations that are vital for life. These unique characteristics arise from the folding of these biomacromolecules into secondary structures, which is enabled by the perfect control over their primary structure. Indeed, the formation of such 3D architectures results in the creation of catalytic pockets that are composed of a set of reactive groups, located in a confined environment. Among other aspects, enhancement of the catalyzed reaction rate can be attributed to the different chemical interactions participating cooperatively, thereby increasing the catalytic activity.^[^ ^195^ ^]^

Mimicking the efficiency displayed by enzymes has proven to be a cumbersome challenge, even though the development of novel catalysts is a topic that has already received widespread attention in both academic and industrial research.^[^ ^195^, ^196^ ^]^ While many attempts have been focusing on polymer‐based systems, the limited control over the monomer order suggests that only a minor fraction of the macromolecular chains actually contains the anticipated active site.^[^ ^54^ ^]^ Consequently, discrete synthetic macromolecules offer a more precise platform for the incorporation of various spatially organized non‐natural building blocks, hence combining the best of both worlds.

A first proof of concept was delivered by Prathap and Maayan, who drastically increased the catalytic activity of a 2,2,6,6‐Tetramethylpiperidin‐1‐yl)oxyl or (2,2,6,6‐tetramethylpiperidin‐1‐yl)oxidanyl (TEMPO)/Cu(I)–phenanthroline/*N*‐methylimidazole (NMI) system that was used for the selective and efficient oxidation of a range of alcohols to their corresponding aldehydes.^[^ ^197^, ^198^ ^]^ By relying on a peptoid backbone as scaffold, they were able to readily screen the influence of the position of two of the involved functional groups (TEMPO and phenanthroline). Intriguingly, placing these two catalytic groups next to one another in the sequence resulted in an increased turnover number (i.e., the number of moles of substrates that are effectively catalyzed by one mole of catalyst), in comparison to their positioning close in space (enabled through folding of the peptoid oligomer, vide infra). Additionally, it was also demonstrated that intramolecular cooperative catalysis did not occur efficiently when using a dimeric system that comprised these catalytic groups. Instead, an additional monomer had to be present in the oligomer (e.g., forming a trimer) in order to obtain high turnover numbers, since the increased sterical hindrance of certain (rotational) conformations forced the catalytic functionalities in close proximity.

Fernandes and co‐workers further improved this catalytic system via the development of a set of novel uniform macromolecules that included all catalytic functionalities, namely TEMPO, NMI, and pyridyltriazole (pyta, the latter was employed as a synthetic alternative to phenanthroline).^[^ ^199^ ^]^ Moreover, these sequence‐defined oligomers were grafted onto mesoporous silica particles, which have been shown to drastically increase the influence of interchain interactions, since the oligomers are now forced into a confined spatial environment. Interestingly, macromolecules bearing the imidazole closer to the copper center (i.e., pyta) showed the highest catalytic activity when grafted onto the surface, yet this gave rise to the lowest turnover number when deployed in a homogenous approach. This apparent discrepancy was rationalized by emphasizing the crucial role that the pyta‐Cu–Imidazole complex played in the catalytic cycle. Oligomers grafted onto the silica particles lack almost all conformational freedom since any type of rotation or back‐folding would induce significant sterical hindrance with the neighboring macromolecules. Therefore, the closer both catalytic sites are located in space, the higher will be chance for the formation of the crucial imidazole–copper center. Moreover, the catalytic center was also formed between different macromolecules, since dilution experiments demonstrated that introducing non‐active PEG oligomers onto the surface significantly reduced the turnover number.

On the other hand, deploying catalytic macromolecules in solution does not suffer from the aforementioned restrictions. Positioning the imidazole moiety further along the chain increases the possibility of the oligomers to fold due to the increase in flexibility and degrees of freedom, which eventually increases the turnover number because the imidazole–copper complex is formed more efficiently. In this case, intrachain interactions become more important than different macromolecules interacting together. Finally, Fernandes and co‐workers demonstrated that the order of catalytic functionalities plays a crucial role in the development of efficient cooperative catalysts.^[^ ^200^, ^201^ ^]^

**Figure 11:**
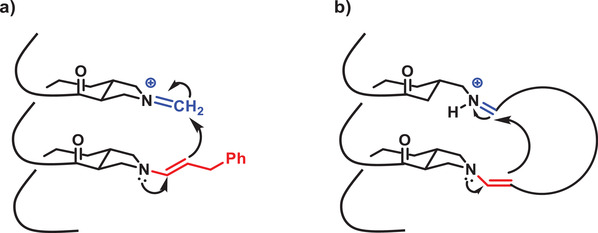
a) Catalysis of a crossed‐aldol reaction involving two aldehydes by two pyrrolidine moieties cooperatively working together. The catalytic groups are positioned together in space through the folding of *β*‐ or *α*/*β*‐peptides. Adapted with permission.^[^ ^202^ ^]^ Copyright 2020, The American Chemical Society. b) The catalyzed cross‐aldol reaction of a bisaldehyde moiety results in the formation of a macrocycle due to the decreased entropic cost. Reproduced with permission.^[^ ^205^ ^]^ Copyright 2019, AAAS.

In another study, Girvin and Gellman screened the dependency of bifunctional catalysis on the geometry of the involved catalytic groups.^[^ ^202^ ^]^ More specifically, catalysis of a crossed aldol reaction between two different aldehydes was reported to occur by two pyrrolidine moieties cooperatively working together, and the influence of the spatial organization of these units on the catalytic efficiency was investigated.^[^ ^203^ ^]^ Relying on *β*‐ or *α*/*β*‐peptides enabled the incorporation of both catalytic groups at different positions along the sequence (**Figure** **11**a), while these peptidomimetics have shown to adopt helical secondary structures that differ significantly (i.e., residues per turn) dependent on the used building blocks.^[^ ^156^, ^204^ ^]^ The authors demonstrated that minimal differences in the sequential order and the geometry of the folded structure tremendously affected the relative rate enhancement. In addition, changing the substrate to a bisaldehyde structure and deploying the best catalytic foldamer resulted in the fast and efficient formation of a cyclic structure (Figure 11b).^[^ ^205^ ^]^

Next to cooperative catalysts, the development of novel asymmetric catalysts that can incorporate a wide range of functionalities could prove to be extremely useful since the majority of building blocks used for the synthesis of pharmaceutical products require absolute control over the stereochemistry. Kirshenbaum and co‐workers were among the first to show that binding an achiral catalyst to a folded peptoid scaffold made it possible to obtain chiral secondary alcohols in a high enantiomeric excess (>99%).^[^ ^190^ ^]^ In this case, the oxidation of either enantiomers from a racemic mixture was favored over the other as a result of the introduction of the asymmetric environment provided by the folded peptoid helix, hence generating an excess of the other, unreactive enantiomer, a process described as oxidative kinetic resolution. Again, it was shown that the position of the active site (TEMPO) into the folded oligomer played a decisive role in the outcome of the catalytic cycle, since minor changes would either invert or completely inhibit the asymmetric catalytic process. In a similar example, Maayan and co‐workers reported the asymmetric catalysis of the Michael reaction between *β*‐pentastyrene and pentanal.^[^ ^206^ ^]^ This was achieved through the development of a novel class of peptoids that exhibited a unique *β*‐turn in their 3D structure, a prerequisite for this specific enantioselective catalysis to reach a high enantiomeric excess. Moreover, it was also shown that the efficiency of the catalytic cycle was in direct correlation with the stability of the secondary structure. For example, when different (rotational) conformations were observed, the enantiomeric excess was significantly reduced, compared to when the desired structure was the most stable one.

In a last example, Izzo and co‐workers promoted the enantioselective benzylation of glycine derivatives by relying on chiral cyclopeptoids as a new class of phase transfer catalysts.^[^ ^207^ ^]^ Even though additional improvements are required to further increase the enantiomeric excess of the final product, these synthetic macrocycles have already proven to be extremely efficient and useful during displacement reactions at an achiral center, outperforming the widely applied crown ethers in some cases.^[^ ^208^ ^]^

These examples all show that uniform macromolecules have the intrinsic ability to mimic the efficiency of enzymes. Moreover, they have the advantage to incorporate numerous synthetic groups, thereby enabling an enormous expansion of the potential reactions that can effectively be catalyzed. Nature has optimized the design of its biomacromolecules according to the “reaction environment” (e.g., water as a solvent, physiological pH, etc.), yet many reactions that are considered indispensable for a synthetic organic chemist do not proceed under similar conditions. For these reasons, we believe that synthetic macromolecules can play an important role in the development of novel catalytic systems, especially when they are composed of multiple functional groups, since these can be precisely positioned along an oligomer in an ideal geometry for catalysis to proceed. Nevertheless, already attaching a single catalytic functionality to a sequence‐defined macromolecule that provides an ideal binding site for the targeted substrate will also significantly enhance the reaction rate constant, as the entropy loss has already been countered by the efficient binding of the substrate.

Although the previous sections were dedicated to applications in material science, many of the examples proposed highlighted the intrinsic link with biology. The following sections will further confirm this link by elaborating on the applications of discrete macromolecules into life science areas.

## 4 Applications of Discrete Synthetic Macromolecules in life Science

Nature makes use of regulated sequences of amino acids in order to make higher order structures such as *α*‐helices or *β*‐sheets that construct peptides or proteins.^[^ ^209^, ^210^ ^]^ The properties of these biopolymers strictly rely on the right sequence of building blocks, thus a focus on sequence regulation has been detrimental in applications with biological importance. Despite the fact that ongoing research is mostly directed toward the use of narrow dispersity polymers made from biologically relevant monomers,^[^ ^211^, ^212^, ^213^, ^214^, ^215^, ^216^, ^217^, ^218^ ^]^ the following section will highlight key examples from recent literature that show different applications of discrete macromolecules in life sciences.

### 4.1 Lectin Binding Behavior of Discrete Oligomers

**Figure 12:**
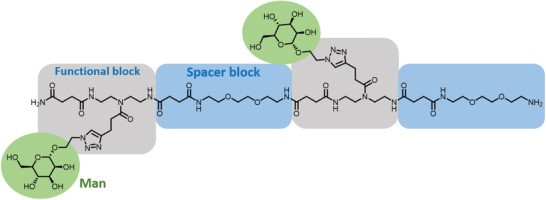
A representative glycomacromolecule. Adapted with permission.^[^ ^231^ ^]^ Copyright 2020, Wiley‐VCH. Typically, examples in the literature employ various chain lengths, different number of carbohydrates or topologies. Additional control can be obtained with different carbohydrate units (green circle), different spacers between binding units (blue box), or different functional blocks tolerating a range of chemistries for carbohydrate conjugation to the backbone, etc. (gray box).

In living matter, carbohydrates play an important role and are crucial for many biological processes including cell recognition, cell growth regulation, adhesion, as well as inflammation and immune responses.^[^ ^219^, ^220^ ^]^ Especially in biomedical treatments, the carbohydrate–cell interaction is required to be highly selective to the targeted cell type, in order to avoid side reactions with other cell, which poses as one of the biggest challenges. The encounter of carbohydrates with a cell takes place over carbohydrate binding proteins (lectins), which are highly abundant on the cell surface.^[^ ^221^ ^]^ Analogous to enzymatic processes in which only specific reaction partners undergo a reaction (lock and key principle), certain carbohydrates are able to interact with lectins explicitly.^[^ ^222^, ^223^, ^224^ ^]^ Moreover, the binding behavior between lectins and carbohydrates is highly specific and selective.^[^ ^225^, ^226^ ^]^ The ability to place sugar units in a predefined order in a macromolecular chain allows to encode biological information (glycocode). As a consequence, it is important to investigate this interaction, in order to understand and decipher the “communication” of carbohydrates with lectins.^[^ ^227^, ^228^, ^229^ ^]^ Since a thorough comparison of every possible combination that includes all lectins and carbohydrates is out of the scope of this work, examples herein will be limited to sequence‐ordered carbohydrate‐containing macromolecules (**Figure** **12**) that are studied with dendritic cell‐specific intercellular adhesion molecule‐3‐grabbing nonintegrin (DC‐SIGN) or Concanavalin A (Con A), which are the most studied lectins in this context.^[^ ^230^ ^]^

An early example was reported by Hartmann and co‐workers, who employed a solid phase approach that resulted in a set of homovalent (single type of carbohydrate) macromolecules containing one, two and three mannose units.^[^ ^232^ ^]^ Whereas the structures were comparable in their overall lengths, the number of binding units and their spacing varied. An increase in Con A affinity was observed with higher valency. Interestingly, the increased spacing in the divalent structure was found to be unfavorable, as it only displayed a moderate affinity increase compared to the monovalent structure. On the other hand, the hydrophilic nature of the flexible backbone was found to be further enhancing binding affinity, in analogy to the trend of hydrophobicity observed for antimicrobial use (Section 4.2). Similar strategies were employed by the same group and others, to prepare heterovalent structures consisting of different binding or non‐binding carbohydrates, which are known to modulate lectin interaction or help to further influence the binding with lectins.^[^ ^233^, ^234^, ^235^ ^]^ For example, Hartmann and co‐workers prepared multivalent chains and coupled them together in a step‐growth polymerization to obtain high molecular weight polymers.^[^ ^236^ ^]^ It should be noted that while such an approach resulted in disperse polymers, the initially obtained chains were monodisperse and locally uniform around the binding units. Nevertheless, a direct dependency between lectin binding ability of the polymers and the number of sugar units could be observed.^[^ ^237^, ^238^ ^]^ Similar outcomes were established with locally monodisperse structures (i.e., in the side chain) that were carried out on brush like glycopolymers.^[^ ^231^ ^]^

Another way to achieve multivalency is via the conjugation of carbohydrates to multifunctional cores, resulting in uniform glycosylated macromolecules.^[^ ^239^ ^]^

While not the scope of this work, it is noteworthy to highlight an example of heterovalent structures that was demonstrated by Nierengarten and co‐workers.^[^ ^240^ ^]^ Herein, the synthesis of a sophisticated supramolecular glycocluster containing ten galactose units on each side of a pillar[5]ene, with two fucose units acting as stoppers for a rotaxanated alkyl chain, was reported. It was shown to achieve high binding affinities toward two separate lectins. Thus, these examples already demonstrate the effect of heterovalency and importance of macromolecular design to elucidate the binding mechanism.

An example of asymmetric branching from a central core was recently reported by Hartmann and co‐workers describing the synthesis of linear and 3‐arm precision glyco‐oligomers employing “click” chemistry on solid phase.^[^ ^241^ ^]^ Moreover, the distance of the binding carbohydrates was varied using various spacers at different lengths resulting in a clear correlation between overall size and inhibitory potential against lectin receptors langerin and Con A. While it is worth noting that this study did not necessarily take valency of the lectins into consideration, the synthesis could have been modified to increase the number of binding units, while for example maintaining an equal overall size. In addition, introduction of heterovalency to these structures could have also allowed for advanced control over binding.

In the above, some key examples are given, which demonstrate the ability to fine tune binding of precisely engineered macromolecules to lectins with the help of precision sequences. So far, these have helped to improve the understanding of factors that directly or indirectly contribute to carbohydrate–lectin interactions, emphasizing the relevance of sequence regulation. However, broader studies that include complex discrete macromolecules with different length, shape and topology against a broad library of lectins are yet to be carried out.

### 4.2 Antibacterial Properties of Discrete Synthetic Macromolecules

While antibiotics drastically increased the life expectancy in the last century, the rise of antibiotic resistant bacteria is one of the biggest risks to human health.^[^ ^242^, ^243^ ^]^ Therefore, scientists have been looking for alternatives to traditional antibiotics.^[^ ^244^ ^]^ In their efforts, they have been inspired by antimicrobial peptides that are expressed by the immune system of all multicellular organisms. These are relatively short peptides, in fact, 88% of the 3180 reported structures contain less than 50 amino acids.^[^ ^69^, ^245^ ^]^ While these peptides show a high structural diversity, many display similar physicochemical properties. In general, they possess a net positive charge resulting from cationic amino acids (e.g., lysine or arginine), but they also possess a large fraction of hydrophobic residues, which is key for their activity.^[^ ^246^ ^]^ Because of their positive charge, they are able to adhere and accumulate semi‐selectively to a bacterial cell membrane, which is richer in negatively charged phospholipids compared to mammalian cells.^[^ ^247^ ^]^ Thus, the insertion of the hydrophobic moieties can potentially disrupt the membrane, thereby killing the bacterial cells.^[^ ^248^, ^249^ ^]^

The use of antimicrobial peptides as antibiotics is hindered by their high production cost and sensitivity toward proteolysis. Therefore, synthetic mimics that replicate these crucial physicochemical properties have been developed.^[^ ^250^, ^251^ ^]^ The antibacterial potency of these mimics is often evaluated by determining the minimum inhibitory concentration (MIC) needed to prevent the proliferation of the bacteria. However, it is also important to assess the toxicity toward eukaryotic cells via, for example, hemolysis assays. It is important to obtain a high selectivity, i.e., high toxicity toward bacterial cells with limited or no toxicity to eukaryotic cells. Design parameters such as architecture, type of cationic group, molecular weight, etc. have been extensively investigated for polymeric systems and are discussed in specialized reviews.^[^ ^252^, ^253^, ^254^, ^255^ ^]^ While polymers with a high hydrophobic content or high molecular weight are often associated with increased toxicity,^[^ ^256^, ^257^ ^]^ sequence‐defined oligomers could help to identify potent antimicrobials. In this context, the precise control of the monomers’ order can be used to further optimize the structure with regard to their antimicrobial effectiveness and selectivity.

**Figure 13:**
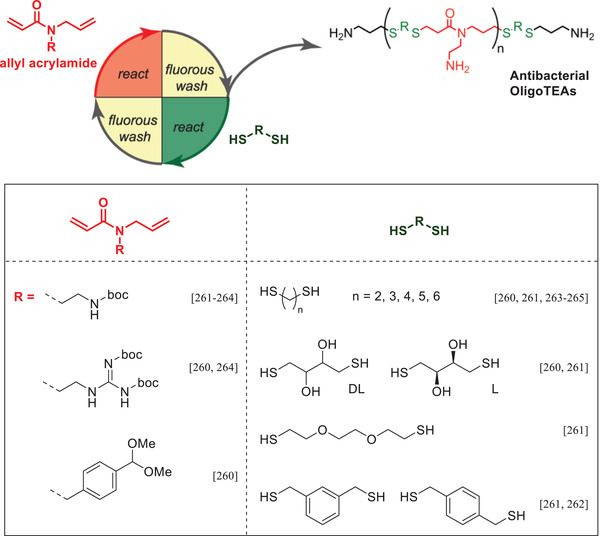
Synthesis of oligoTEA's using allyl acrylamide building blocks and different dithiols. Adapted with permission.^[^ ^260^ ^]^ Copyright 2017, The American Chemical Society.

Porel and Alabi reported the preparation of sequence‐defined oligothioeteramides (oligoTEA) with amine and guanidinium side chains, which were shown to have antibacterial properties.^[^ ^28^, ^258^, ^259^, ^260^, ^261^, ^262^, ^263^, ^264^ ^]^ Synthesis of these oligomers relies on functionalized allyl acrylamide building blocks and dithiols (**Figure** **13**). The acrylic moiety readily reacts with thiols in a phosphine‐catalyzed Michael addition, while the allyl moiety is inert under these conditions. This orthogonality is exploited to synthesize the oligomers in a stepwise iterative procedure. To facilitate purification in between each reaction step, a fluorous tag was used, which was then removed after synthesis.

In their first study, macrocyclic oligoTEA's with up to three guanidinium moieties (Figure 13) were investigated.^[^ ^259^ ^]^ For that, a linear oligoTEA was first synthesized and cleaved from the fluorous tag at the final stage before being cyclized in a single one‐pot reaction. This was possible by using an elegant approach combining a clever choice of protective groups and cyclisation strategy. All reported linear and macrocyclic oligoTEA's showed antimicrobial activity, with MIC values between 3.5 and 31.2  × 10^−6^ m toward *Escherichia coli* (*E. coli*) bacteria. In comparison, the commonly used antibiotic ampicillin had a MIC of 44.2 × 10^−6^ m in the same assay, indicating the potency of these synthetic macromolecules. The cyclisation of the oligoTEA's enhanced their antimicrobial activity, a trend that was also observed for other macrocycles.^[^ ^265^ ^]^ This effect is often attributed to the reduction in mobility upon cyclization, which can assist in the adoption of an amphiphilic conformation required for their antimicrobial role. 2D ^1^H NMR exchange spectroscopy indicated that the macrocycles can switch between different conformations but become more rigid when the ring size is decreased. In general, smaller macrocycles performed better, whereas the replacement of guanidinium moiety with a butyl group, showed a decrease in the antibacterial potency. An interesting feature of this chemistry is the ability to change the polarity of the backbone by using different dithiols (Figure 13). Remarkably, when one of the propyl groups on the backbone was substituted with the more hydrophilic dl‐dithiothreitol, no difference in the antimicrobial activity was observed. On the other hand, this modification led to a decrease in hemotoxicity of the latter compound.

In subsequent studies, the effects of the chain length, the hydrophilic/hydrophobic backbone moieties, and the effect of the sequence were investigated with linear oligoTEA's.^[^ ^260^, ^261^, ^262^ ^]^ A library of different tetramers bearing primary amine side chains that differed in backbone polarity was screened.^[^ ^260^ ^]^ The overall hydrophobicity was quantified by the retention time in reversed‐phase high performance liquid chromatography (HPLC) and correlated with the antimicrobial potency of the oligomer. Highly hydrophilic oligomers showed only little activity toward *Bacillus subtilis* and *Staphylococcus epidermidis* bacterial strains. However, increasing the hydrophobicity above a certain threshold leads to a pronounced increase in antimicrobial properties with only a few exceptions. On the other hand, a hemolysis assay demonstrated that the toxicity gradually increased with the overall hydrophobicity. Nevertheless, it was found that the local hydrophobicity (i.e., the sequence of hydrophobic groups) could be tuned to optimize the antibacterial potency.^[^ ^262^ ^]^ In this context, aliphatic C2, C3, and C5 dithiol building blocks (Figure 13) were used to synthesize tetramers. Rearrangement of these groups did not affect the overall hydrophobicity of the oligomer, but local differences arise due to the length of the dithiol. While all these oligomers had a similar toxicity, the antibacterial activity showed clear differences. One of them was roughly five times more potent, thereby indicating that the sequence could be used to optimize their activity. These studies confirm that a fine balance between cationic charges and hydrophobic groups is essential to both obtain antimicrobial activity in combination with low toxicity to eukaryotic cells, but the sequence of the groups can also be used to further fine‐tune the properties.

**Figure 14:**
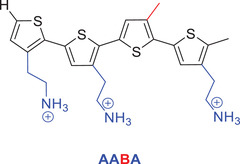
Example of an oligo(thiophene)s structure with primary amine moieties A) and inert B) spacer unit, studied by Palermo and co‐workers.^[^ ^267^ ^]^

Palermo and co‐workers investigated sequence‐defined oligo(thiophene)s with multiple primary amine moieties in the side chain, which were synthesized by iterative organometallic coupling reactions (**Figure** **14**).^[^ ^266^, ^267^ ^]^ These oligomers show antibacterial activity, but their potency is further increased under visible light because of their dual role. Besides binding to the bacterial cell membrane, they can also generate reactive oxygen species under visible light, which further contribute to their activity. In this study, it is also observed that the chain length, as well as the sequence order, influences the antibacterial activity. Also, more hydrophilic oligomers induced less hemolysis. Interestingly, two isomeric trimers had the same potency in the dark but displayed a 6× fold difference under visible light. This indicates that the binding and disruption of the membrane is not very sensitive toward changes in the sequence of these short oligomers, but the production of reactive oxygen species seems to be affected.

To investigate the effect of dispersity, the authors prepared an equimolar mixture of three alternating oligomers (AB, ABAB, and ABABAB). Thus, the average length corresponds to that of a tetramer, resulting in a theoretical polydispersity of 1.17. Remarkably, the potency of this mixture was four times lower than the individual compound (ABAB). While the observed MIC of the mixture followed the predictions based on the individual compounds, it was found to be significantly lower compared to the individual compounds. This study shows that dispersity can have a significant influence for such applications, as the disperse nature of the sample can mask the effect of a very potent oligomer or polymer.

These examples demonstrate the contribution of sequence‐defined macromolecules to the field of synthetic antimicrobials to identify structures with a high antimicrobial potency, while the control over the sequence can be used to further fine tune their properties.^[^ ^268^ ^]^

## 5 Other Applications of Discrete Synthetic Macromolecules

In addition to the described examples, there are further reports that involve the use of sequence‐defined oligomers, which cannot be categorized in one of the above‐mentioned categories. A selection will be briefly presented in the following.^[^ ^269^ ^]^

For example, Meier and co‐workers applied the iterative synthesis concept of sequence‐definition to conjugated, rod‐like pentamers based on oligo(phenylene ethynylene)s.^[^ ^17^ ^]^ Although the resulting macromolecules showed only a slight difference in photophysical properties, the variation was more profound in the hydrodynamic volume and thermal properties. The study could be extended to longer sequences beyond a pentamer that could additionally influence the photophysical properties. However, despite the encouraging results, the macromolecules being obtained in very low overall yields (i.e., 3.2%), new synthetic strategies need to be developed for the use of fully conjugated macromolecules in real‐world electronic applications.

Börner and co‐workers reported a series of studies that used a number of synthetic, sequence‐defined peptidomimetics for drug delivery purposes.^[^ ^270^, ^271^, ^272^ ^]^ Possible candidates from a large library were prepared by combinatorial strategies adapted from a single‐bead single‐compound concept. Further investigation of the drug‐interacting oligomers revealed a strong dependency of both drug‐payload capacities and release kinetics on the oligomers’ sequence.^[^ ^270^ ^]^ In a recent report, peptidomimetic synthetic macromolecules were able to encapsulate 40% more of the same drug payload and show a higher sensitivity to release kinetics by simply varying the side chains.^[^ ^271^ ^]^ In a similar study, a synthetic macromolecule was shown to reach a higher drug loading capacity (i.e., 69%), while retaining similar properties compared to the parent peptide.^[^ ^272^ ^]^ The ability to encapsulate dye molecules by uniform star‐shaped block‐macromolecules was likewise demonstrated by Meier and co‐workers.^[^ ^273^ ^]^

Using a peptoid‐based backbone, Bräse and co‐workers showed that implementing different side chains improved the cell uptake of a Rhodamin B dye.^[^ ^274^ ^]^ For this purpose, a range of positively charged functionalities were used that contained either a crown ether moiety or multiple amino groups in the side chain. Their incubation with HeLa cells allowed to visualize the uptake of these sequences via confocal fluorescence microscopy, which revealed that the hexamers resulted in different intracellular distributions. While it was possible to predict the likely aim of a certain sequence within the cell, a trend with regard to the influence of the cationic side chains on the target could not be established. In a similar fashion, Alabi and co‐workers demonstrated the possibility to alter hydrophilicity, antigen binding and in vitro potency of antibody–drug conjugates by simply varying the distance between each other.^[^ ^275^ ^]^ For this purpose, pegylated sequences of trimers were employed to place a cytotoxic drug at different distances from the antibody, resulting in three isomeric antibody‐drug conjugates. The same group used a similar strategy, where a sequence with a pH‐sensitive bond was employed, to study bond degradation within living cells.^[^ ^276^ ^]^ The monodispersity effect of PEG based low‐fouling coatings was investigated recently, in a comparative study by Cui and co‐workers. The non‐specific interactions with cells and proteins could be minimized, when monodisperse PEG at low molecular weight was used (752 Da), whereas a molecular weight average of 2000 Da was required for PEG of disperse nature.^[^ ^277^ ^]^

These examples that usually employ a single variation along a sequence‐defined oligomer do not only help to understand key interactions but also reveal some of the unknown aspects of biological processes.

## 6 Conclusion and Future Trends

The field of precisely engineered, discrete macromolecules is wide in scope as highlighted by the range of applications presented in this report. With emerging elegant synthetic routes that enable the transition from disperse polymeric systems to ultraprecise macromolecules, the scientists will be able to obtain even more complex structures that will broaden our range of applications. Especially in life sciences, mimicking biological systems continues being a starting point, which is aimed to evolve into complex, machine‐like systems that operate in living organisms and is expected to potentially allow for tailor‐made and programmable therapies. The flagship in material sciences is expected to continue with data storage and encryption applications. This area of research is highly present in the recent studies but yet, more research is required in order to translate the current fundamental know‐how into routine applications. In addition, technologies that will allow for rewriting/deleting or fast reading of molecular information should be developed or improved, as they are still at their infancy stage. For all real‐life applications, their success will be related to the synthetic approaches being fast, high yielding, error‐free and with the potential to be automated. Surely, other applications will benefit from these developments in the coming years.

With regard to scalability and chain‐length aspects, some more efforts are still required to allow for facile high scale synthesis of long discrete macromolecules in the near future. All these improvements will pave the way to untapped realms of research questions that scientists have never even thought of asking before. To our opinion, the exploration of applications for discrete macromolecules is only starting and future possibilities seem to be endless.

## Conflict of Interest

The authors declare no conflict of interest.
